# Complete Protection Against *Yersinia pestis* in BALB/c Mouse Model Elicited by Immunization With Inhalable Formulations of rF1-V10 Fusion Protein *via* Aerosolized Intratracheal Inoculation

**DOI:** 10.3389/fimmu.2022.793382

**Published:** 2022-01-26

**Authors:** Wei Zhang, Xiaolin Song, Lina Zhai, Jianshu Guo, Xinying Zheng, Lili Zhang, Meng Lv, Lingfei Hu, Dongsheng Zhou, Xiaolu Xiong, Wenhui Yang

**Affiliations:** State Key Laboratory of Pathogen and Biosecurity, Beijing Institute of Microbiology and Epidemiology, Beijing, China

**Keywords:** *Yersinia pestis*, pneumonic plague, subunit vaccine, rF1-V10, dry powder formulation, aerosolized intratracheal inoculation, mucosal immune response

## Abstract

Pneumonic plague, caused by *Yersinia pestis*, is an infectious disease with high mortality rates unless treated early with antibiotics. Currently, no FDA-approved vaccine against plague is available for human use. The capsular antigen F1, the low-calcium-response V antigen (LcrV), and the recombinant fusion protein (rF1-LcrV) of *Y. pestis* are leading subunit vaccine candidates under intense investigation; however, the inability of recombinant antigens to provide complete protection against pneumonic plague in animal models remains a significant concern. In this study, we compared immunoprotection against pneumonic plague provided by rF1, rV10 (a truncation of LcrV), and rF1-V10, and vaccinations delivered *via* aerosolized intratracheal (i.t.) inoculation or subcutaneous (s.c.) injection. We further considered three vaccine formulations: conventional liquid, dry powder produced by spray freeze drying, or dry powder reconstituted in PBS. The main findings are: (i) rF1-V10 immunization with any formulation *via* i.t. or s.c. routes conferred 100% protection against *Y. pestis* i.t. infection; (ii) rF1 or rV10 immunization using i.t. delivery provided significantly stronger protection than rF1 or rV10 immunization *via* s.c. delivery; and (iii) powder formulations of subunit vaccines induced immune responses and provided protection equivalent to those elicited by unprocessed liquid formulations of vaccines. Our data indicate that immunization with a powder formulation of rF1-V10 vaccines *via* an i.t. route may be a promising vaccination strategy for providing protective immunity against pneumonic plague.

## 1 Introduction

*Yersinia pestis*, a non-motile, facultative intracellular, gram-negative bacterium, is the causative agent of plague (1–3). Plague, a devastating zoonotic disease prevalent in many parts of the world, is transmitted through infected fleas from rodent reservoirs to humans (4, 5). It is estimated to have claimed over 200 million human lives during the course of three major human pandemics (6). The most recent outbreak in Madagascar (2017–2018) resulted in ~2400 cases and ~200 deaths, raising global concerns (4).The control of human plague outbreaks relies mainly on the rapid confirmation of the diagnosis, isolation and treatment of confirmed and suspected cases (5). For confirmed patients, effective antibiotics (such as tetracycline, streptomycin, chloramphenicol) and preventive therapies must be administered within 24 hours of the onset of symptoms (7). Especially, pneumonic plague transmitting through aerosol droplets is the most dangerous among the three primary clinical forms due to its rapid onset and progression (6, 8). Without the rapid response with appropriate antibiotics, the fatality rate of pneumonic plague approaches 100% (9). Moreover, *Y. pestis* remains listed as a Tier 1 Select Agent because of its potential use as a biological weapon in an aerosolized form, making it an urgent public health and safety priority (10, 11). Therefore, development of a protective vaccine that provides both rapid and long-lasting immunity in the event of mass exposure to aerosolized *Y. pestis* is of great interest.

Historically, killed whole-cell vaccines (KWCVs) and live whole-cell vaccines (LWCVs) have been successfully used to protect humans against plague in parts of the world (12). To prepare KWCVs, *Y. pestis* were inactivated by heating or with chemicals. These vaccines evoked immunity against bubonic plague but were inefficient against pneumonic plague in animal models (13, 14). KWCVs are no longer used due to questionable efficacy and considerable reactogenicity. LWCVs were prepared from fully virulent strains of *Y. pestis* after multiple passages. The former Soviet Union and other nations still use LWCVs for human vaccination, e.g., the NIIEG line of the *pgm*-negative strain EV76. LWCVs are able to protect humans against bubonic and pneumonic plague (15–17), however, these vaccines are associated with several adverse effects, and fail to provide long-term immunity (1, 18). In addition, safety concerns have limited enthusiasm for the development of LWCVs and they are only recommended in endemic areas (19).

The subunit vaccine provides the most promise as a plague vaccine. Development efforts for an effective subunit vaccine to pneumonic plague have focused on two primary antigens of *Y. pestis*, namely the capsular protein (F1) and the low calcium response protein (LcrV). Baker et al. (20) first purified F1 protein and demonstrated that a vaccine with F1 protected mice and rats from bubonic plague. However, the F1 vaccine candidate provided only 65-84% protection against pneumonic plague (21); vaccines based exclusively on F1 were ineffective against F1-negative *Y. pestis*, which may be as virulent as wild-type (WT) CO92 *Y. pestis* (22). Burrows (23) discovered that LcrV was an important virulence protein and subsequent studies confirmed it was a critical protective antigen against *Y. pestis* infection (19, 24, 25). Unfortunately, part of the LcrV protein, acid residues 271-300, partially suppresses host immune response by stimulating interleukin-10 (IL-10), which suppresses Th1 cells (26, 27); this limits its usefulness in vaccines. The combination of recombinant F1 and LcrV antigens (rF1-LcrV) has a good safety profile in various animal models (28, 29), elicits greater protection than either F1 or LcrV alone (30, 31), but rF1-LcrV does not confer complete protection for mice challenged with more than 255× LD_50_ *Y. pestis* administered *via* inhalation (32–34). Most recently, plague vaccines based on the expression of protective antigens of *Y. pestis* in live vectors (bacterial or viral platform) were developed (35) but had obvious limitations. There is thus a need to continue research on subunit vaccine candidates, which require further modification to minimize shortcomings and elicit more robust immune protection against pneumonic plague.

Over the past few decades, pulmonary delivery of vaccines has received increasing attention due to its ability to recruit local immune responses of the bronchopulmonary mucosa in addition to the broader systemic immune response (36–38). In addition, administration of vaccines *via* the lungs shows better bioavailability and more rapid effectiveness than injection routes because of the lung’s large surface area, abundant blood flow, and highly permeable epithelium (39, 40). Currently, there are two formulations of inhalable vaccines: (i) liquid formulations that require a cold chain transport system to maintain vaccine potency; and (ii) powder formulations that have long-term stability at room temperature for storage and shipping (41, 42). Given its obvious advantages, the latter is attracting more attention for use in aerosolized intratracheal (i.t.) delivery of vaccines. For more than 70 years, the *Y. pestis* EV NIIEG strain has been used as a human plague vaccine in the former Soviet Union and confers protection against bubonic and pneumonic plague after administration *via* inhalation (16, 43, 44). However, the protection appears to be short-lived and the vaccine is highly reactogenic, limiting licensing of this vaccine for use in many parts of the world (1, 12, 45). The preparation of live *Y. pestis* dry powder is rarely reported in the literature, possibly because of bacterial viability being lost during preparation. Subunit vaccine candidates may thus prove a better option for inhalable powder. In this study, we improve the immunoprotection of subunit vaccines against pneumonic plague by preparing the rF1, rV10 (a truncation of LcrV), or rF1-V10 fusion protein using spray freeze drying (SFD) to generate dry powder with the adjuvant CpG for i.t. inoculation. We then explore the immunogenicity and protective efficacy of these three subunit vaccines in different formulations (liquid, powder and reconstituted powder) *via* i.t. and subcutaneous (s.c.) administration routes in a mouse model of *Y. pestis* i.t. infection. Our results demonstrate preclinical feasibility of using a powder formulation of rF1-V10 and the potential use of an alternative pulmonary delivery method.

## 2 Materials and Methods

### 2.1 Expression and Purification of rF1, rV10, and rF1-V10

**Figure 1:**
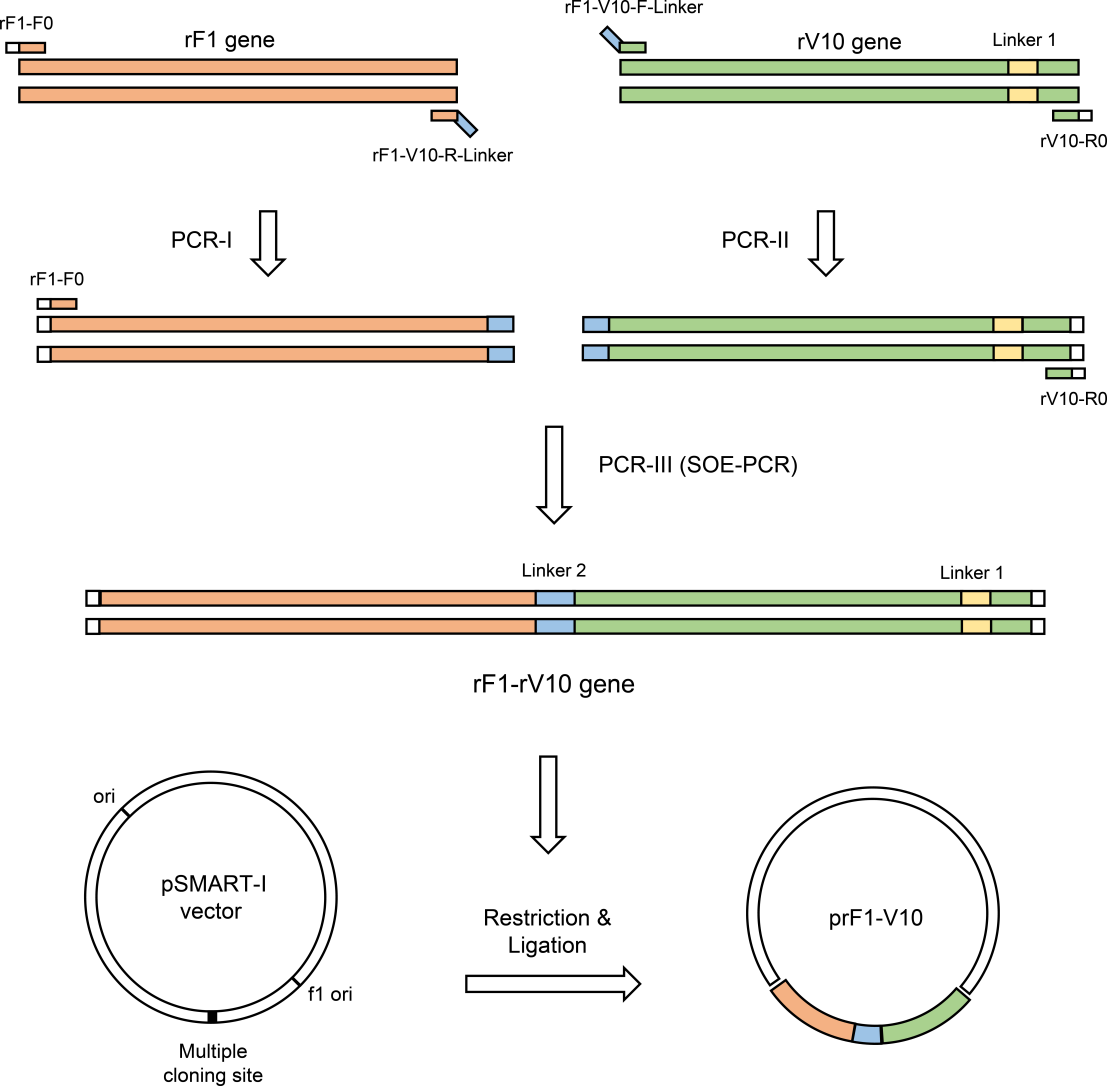
Schematic representation of PCR amplification, splicing and cloning of chimeric rF1-V10 gene in pSMART-I vector.

Recombinant plasmids were built as shown in **Figure 1**. The primers used for DNA synthesis are listed in **Supplementary Table 1**. The DNA sequence encoding rF1 (F1_22-170_, Accession No.: NC_003134) without signal peptide (46) was amplified by PCR. Previous studies have reported that rV10, lacking amino acid residues 271 to 300 (LcrV_271-300_), lost the ability to induce IL-10, significantly reducing its immunosuppressive properties on mice compared to the intact LcrV (27, 47). Therefore, in our study, the sequence encoding the immunosuppressive fragment of LcrV (LcrV_271-300_, Accession No.: NC_003131) was removed and the DNA sequences encoding LcrV_1-270_, the linker Gly-Thr dipeptide, and LcrV_301-326_ were fused in sequential order to obtain rV10 by overlap extension PCR. In brief, the DNA fragments of LcrV_1-270_ and LcrV_301-326_ were generated using primer pairs (rV10-F0, rV10-R0) and (rV10-F, rV10-R; these contain the DNA sequence of the linker) in the first PCR; these two DNA fragments were subsequently used as templates in the second PCR with rV10-F0 and rV10-R0 primers to generate rV10. To obtain the rF1-V10 sequence, DNA sequences encoding rF1, the linker Glu-Phe dipeptide, and rV10 were fused in sequential order by overlap extension PCR, and the two PCR products (rF1 and rV10) generated using primer pairs (rF1-F0 and rV10-R0) and (rF1-V10-F and rF1-V10-R; these contain the DNA sequence of the linker) in the first PCR. The rF1-V10 were generated by a second PCR using primer pairs rF1-F0 and rV10-R0. Finally, the DNA fragment rF1, rV10, or rF1-V10 was inserted into the *BamHI/XhoI* digested pSMART-I (Ming Chen Zhi Yuan Biotechnology Co. LTD., Beijing, China) plasmid by In-Fusion cloning to generate pSMART-rF1, pSMART-rV10, or pSMART-rF1-V10 vectors, respectively.

These three recombinant proteins were purified by affinity chromatography from lysates of *E. coli* BL21 cells (Novagen, Madison, WI, USA) following a previously published protocol (27). The His-Sumo tags at the N terminus of the purified proteins were excised by Sumo Protease (ULP403) and the resulting proteins were analyzed by SDS-PAGE and Western blot. Protein concentration was analyzed using the bicinchoninic acid (BCA) method (Pierce Biotechnology; Rockford; IL). Endotoxin level was assayed using *Limulus* amebocyte lysate (QCL-1000; Cambrex, NJ) and found to be 10 Endotoxin Units (EU) per 1 mg purified protein (48).

### 2.2 Preparation of Powder Formulations of the rF1, rV10, and rF1-V10 Vaccines

The subunit vaccine powders were prepared by SFD as described in published literature (49, 50). Specifically, each subunit vaccine was dissolved in spray drying solution containing D-mannitol, myo-inositol, L-leucine, and poloxamer 188 as excipients and CpG as a mucosal adjuvant (51). This solution was kept in an ice bath and then sprayed into a vessel of liquid nitrogen with a two-fluid pneumatic spray nozzle (2 mm diameter; TSE Systems GmbH, Berlin, Germany) using a fixed back pressure of 1.5 bar and a liquid feed rate of 5 ml/min. The sprayed atomized droplets were quickly frozen into ice crystals under liquid nitrogen. After liquid nitrogen was evaporated, the ice crystals were transferred to a stainless-steel vessel followed by lyophilization in a vacuum freeze-drying system for 48 h at a manifold temperature of -55°C and a vacuum pressure of 10 Pa. The obtained powders were stored at 4°C until further use.

To evaluate the quality and stability of the dry powder formulation, these subunit vaccines were reconstituted in deionized water, then analyzed by SDS-PAGE and Western blot (using sera collected from immunized mice). The volume median diameter (VMD) of dry powder vaccines was measured using a laser particle size analyzer (RODOS & HELOS, Sympatec, Clausthal-Zellerfeld, Germany) and the mass median aerodynamic diameter (MMAD) of the vaccine aerosol particles was measured with the aerodynamic particle sizer (APS) spectrometer 3321 (52) (TSI Inc., St. Paul, MN). The moisture content of vaccine dry powders was determined using thermogravimetric analysis (TGA), as described previously (50). Particle morphology was examined with a Hitachi S-3400N scanning electron microscope.

### 2.3 Animals

Female BALB/c mice (SPF) at 6-weeks of age were obtained from Charles River Laboratories (Beijing, China). This study was approved by the Institute of Animal Care and Use Committee (IACUC) at the Academy of Military Medical Sciences (AMMS), ethical approval number IACUC-DWZX-2021-057.

### 2.4 Bacteria Strain and Growth Media

*Y. pestis* strain 201, which is avirulent to humans (53), is maintained in our laboratory. It was cultivated in Brain Heart Infusion broth (BHI; BD, Voigt Global Distribution Inc., Lawrence, KS). Cultures were inoculated overnight with BHI (dilution 1:20) and cultured at 26°C in a shaking incubator at 220 rpm to an optical density at 600 nm (OD600) of ~1.0. Cultures were then inoculated with BHI (dilution 1:100) and maintained at 26°C, again to OD600 ~1.0. After that, cultures were transferred to a 37°C shaking incubator for another 3 h. For growth on a solid surface, *Y. pestis* was grown on 5% sheep blood agar (SBA) plates (Luqiao, Beijing, China) at 26°C for 3 days.

### 2.5 Immunization and Challenge

**Figure 2:**
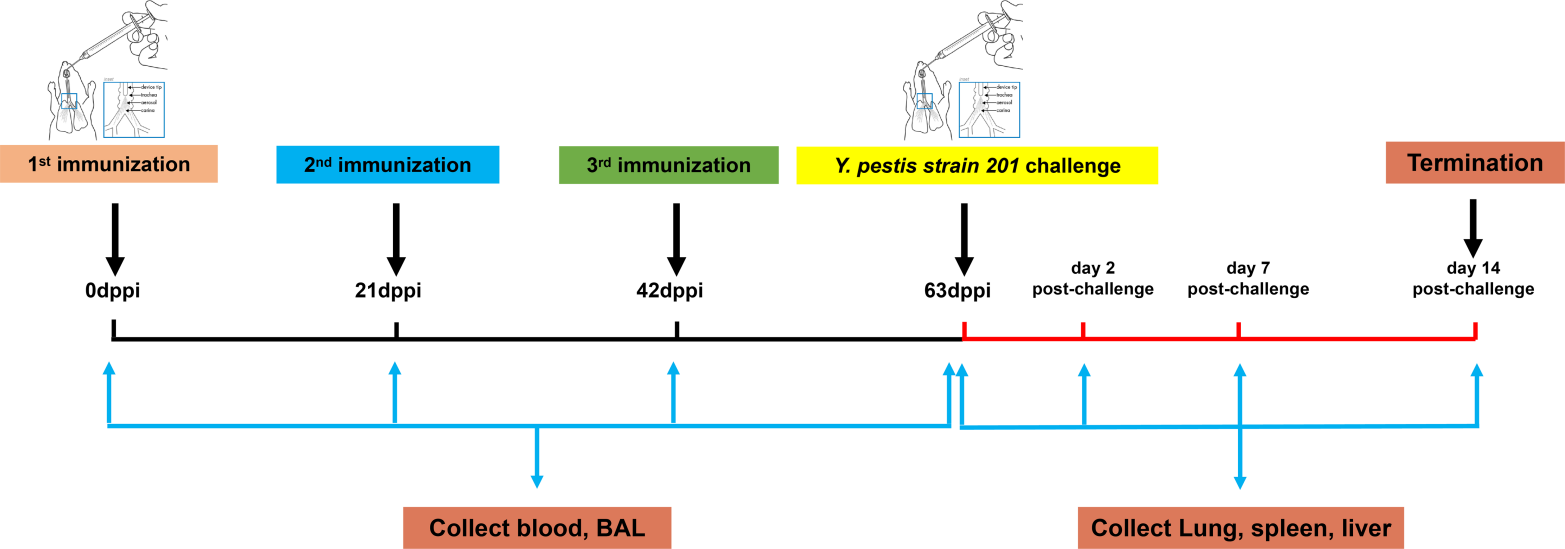
Schema of the immunization protocol. BAL, bronchoalveolar lavage; dppi, days post-primary immunization.

Mice (41 per group) were immunized three times at 3-week intervals (days 0, 21, and 42) through i.t. or s.c. routes (**Figure 2**). The i.t. immunization was performed using a Dry Powder Insufflator for powder inoculation and a MicroSprayer Aerosolizer for reconstituted powder or liquid inoculation following previously described methods (54). Group details are shown in **Table 1** (five experimental groups per protein, five negative controls, and two blank controls). For rF1, the five experimental groups included three groups of mice i.t. inoculated with 1) 0.5 mg of rF1 dry powder (i.t.-rF1, powder), 2) 0.5 mg of rF1 dry powder reconstituted in PBS (i.t.-rF1, reconstituted powder) to create a reconstituted solution, designed as a quality control to assess the influence of the SFD process on vaccine efficacy, and 3) 20 μg of rF1 liquid without SFD (i.t.-rF1, liquid) in PBS. Since the powder vaccine is not suitable for use in s.c. injection, the next two groups of mice were inoculated *via* s.c. with 4) 0.5 mg rF1 dry powder reconstituted in PBS (s.c.-rF1, reconstituted powder), or 5) 20 μg of rF1 liquid without SFD (s.c.-rF1, liquid). The same five experimental group treatments were used for rV10 and rF1-V10 vaccines. Mice in negative controls were immunized with CpG and those in blank controls were immunized with PBS.

**Table 1:** Summary of immunization groups used in the experiment.

Number	Group	Immunization route	Formulation type	Antigen dose (μg/mouse)	CpG dose (μg/mouse)	Volume (μl/mouse)	
1	rF1	i.t.	powder	20	20	50	
2			powder reconstituted	20	20	50	
3			liquid	20	20	50	
4		s.c.	powder reconstituted	20	20	100	
5			liquid	20	20	100	
6	rV10	i.t.	powder	20	20	50	
7			powder reconstituted	20	20	50	
8			liquid	20	20	50	
9		s.c.	powder reconstituted	20	20	100	
10			liquid	20	20	100	
11	rF1-V10	i.t.	powder	20	20	50	
12			powder reconstituted	20	20	50	
13			liquid	20	20	50	
14		s.c.	powder reconstituted	20	20	100	
15			liquid	20	20	100	
16	CpG	i.t.	powder	/	20	50	
17			powder reconstituted	/	20	50	
18			liquid	/	20	50	
19		s.c.	powder reconstituted	/	20	100	
20			liquid	/	20	100	
21	PBS	i.t.	liquid	/	/	50	
22		s.c.	liquid	/	/	100	

i.t., aerosolized intratracheal inoculation; s.c., subcutaneous injection.

At 63 days post-primary immunization (dppi), immunized mice were anesthetized by intraperitoneal injection of pentobarbital sodium and then challenged with 50× LD_50_ (1,000 CFU) or 1,000× LD_50_ (20,000 CFU) of *Y. pestis* strain 201 through an i.t. route using a MicroSprayer Aerosolizer. The direct inhalation of aerosolized *Y. pestis* was used to better mimic the natural aerosol infection of pneumonic plague. Animals were checked daily for morbidity and mortality over the course of 2 weeks. At days 2, 7, and 14 post-challenge, bacterial load analysis was done for the low-dose challenge groups, three mice per group were sacrificed. The lungs, spleens, and livers of these mice were harvested individually and approximately 100 mg of each organ homogenized in 800 µl PBS. Samples of 50 µl of homogenate were diluted serially in PBS, and 10 µl dilutions were plated onto blood agar plates for bacterial counting as previously described (54).

### 2.6 ELISA Assay of Specific Antibody

Sera and bronchoalveolar lavage (BAL) from four mice per group were collected after each immunization (at 21, 42 and 63 dppi). The titers of IgG (sera and BAL) and IgA (BAL) antibodies against the cognate recombinant proteins were evaluated by enzyme-linked immunosorbent assay (ELISA) as described in previous studies (55). Briefly, individual wells in 96-well plates were coated with rF1, rV10, or rF1-V10, plates were blocked with bovine serum albumin (BSA) and incubated with serially diluted sera collected from cognate antigen immunized mice. After washing, the plates were incubated with HRP-conjugated goat anti-mouse IgG or IgA (Abcam, Cambridge, MA). 3,3’,5,5’-tetramethylbenzedine (TMB) was used as substrate, and optical density (OD) was measured at 450 nm with a reference filter (630 nm).

### 2.7 ELISPOT Assay

At 63 dppi, total mononuclear cells were isolated from spleens of three mice per group and suspended (1×10^7^·ml^-1^) in DMEM basic medium (Gibco, Shanghai, China) containing 10% (v/v) fetal bovine serum (Gibco, Australia) and 1% (v/v) penicillin–streptomycin (Gibco, Grand Island, USA). The cells were then plated into a 96-well ELISPOT plate (Mabtech, Nacka Strand, Sweden) with 1×10^6^ cells per well, as previously described. The cells in each well were stimulated with 5 µg of each cognate protein (rF1, rV10, or rF1-V10), Concanavalin A (ConA, positive control, Sigma-Aldrich, Germany), or cell culture medium (negative control) and then incubated for 20 h at 37°C under 5% CO_2_. The levels of IFN-γ or IL-4 were measured by ELISPOT assays as previously described (56).

### 2.8 Histopathology

Animal tissues from three mice per group were collected at 63 dppi and day 2 post-challenge, fixed in 4% paraformaldehyde and embedded in paraffin. Blocks were cut into 5-μm sections, which were stained with hematoxylin and eosin (HE). Pathological alterations in tissue slices were observed by light microscopy. Tissue sections were evaluated blind by a trained pathologist according to the following scores: 0, no pathological lesions; 1, minimal; 2, mild; 3, moderate; 4, severe.

### 2.9 Statistical Analysis

Data are expressed as mean ± SEM. All statistical analyses were performed using SAS statistical software (version 9.1, SAS Institute Inc., Cary, NC) or GraphPad Prism 8.0. Differences in the levels of antibodies and bacterial load among all groups of mice were tested using a two-way ANOVA, followed by LSD analysis or Tukey’s test. IFN-γ and IL-4 levels were compared by one-way ANOVA, followed by LSD analysis. Mouse survival rate was analyzed using Kaplan–Meier survival estimates. *P* < 0.05 was considered significantly different for all statistical analyses.

## 3 Results

### 3.1 Preparation and Characterization of Pulmonary Delivery Vaccines

**Figure 3:**
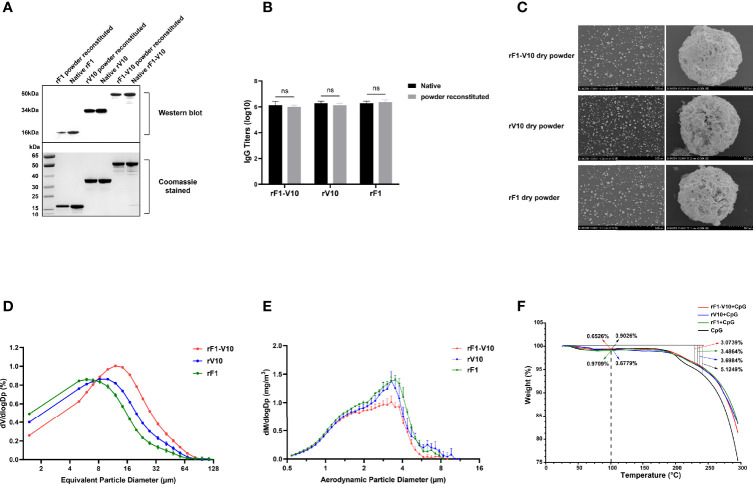
Characterization of rF1, rV10 and rF1-V10 dry powders. **(A)** Dry powders and liquids of three vaccines were analyzed by Western blot using sera collected from cognate antigen immunized mice (upper panel). A protein gel stained with Coomassie brilliant blue was used as a loading control (lower panel). **(B)** The immunogenicity of dry powder and liquid vaccines measured by ELISA, using sera collected from cognate antigen immunized mice. **(C)** Scanning electron microscopy pictures of the three vaccine dry powders. **(D)** VMD of the aerosolized vaccine dry powders, as determined using a laser particle size analyzer. **(E)** MMAD of the aerosolized vaccine dry powders, as measured using a TSI APS 3321. **(F)** TGA of the three vaccine dry powders. ns, no statistical significance.

Following preparation, dry powder vaccines were characterized for structural integrity, immunogenicity, morphology, uniformity of distribution, residual moisture content, and aerodynamic parameters. Molecular weights of reconstituted rF1, V10, and rF1-V10 powders were identical to those of the liquid formulation (~16, ~34, and ~50 kDa, respectively; **Figure 3A**). Both reconstituted powder and the liquid formulations reacted to sera from cognate antigen immunized mice, and titers did not differ significantly (**Figures 3A, B**). The results demonstrate that biochemical integrity and immunogenicity of the three vaccines were unaffected by the SFD process.

Scanning electron micrographs (SEM) of the three vaccine dry powders (rF1-V10, rV10, and rF1) are shown in **Figure 3C**. As expected, the particles were spherical and porous. Three random areas in an SEM graph of the dry powder were chosen for analysis. The VMD of the three vaccine powders, as determined by a laser particle size analyzer, were 7.85 ± 0.68 µm (rF1), 9.75 ± 0.33 µm (rV10), and 13.16 ± 0.27 µm (rF1-V10) (**Figure 3D**). Moreover, the MMAD of aerosol particles, as measured by APS spectrometer 3321, were 2.45 ± 0.05 µm (rF1), 2.45 ± 0.09 µm (rV10), and 2.17 ± 0.06 µm (rF1-V10) (**Figure 3E**). The residual moisture content of the vaccine dry powders was determined by TGA to be approximately 0.971% (rF1), 0.678% (rV10), and 0.653% (rF1-V10) (w/w) (**Figure 3F**). The results indicate that all three vaccine dry powders prepared were suitable for aerosol inhalation.

### 3.2 Systemic Humoral and Lung Mucosal Immune Responses Induced by rF1, rV10 and rF1-V10 Vaccines *via* i.t.

Antigen-specific IgG in sera and antigen-specific IgG and secretory immunoglobulin A (SIgA) in BAL were evaluated after delivery of the three subunit vaccines (rF1, rV10 and rF1-V10) with different formulations (liquid, powder, and reconstituted powder) and *via* different routes (i.t. and s.c.). The antibodies against native F1 or LcrV were not tested in this study, because extractions of these proteins require a laborious and time-consuming processes and result in low efficiency and purity.

#### 3.2.1 Systemic Humoral Immune Responses Induced by Vaccines *via* i.t.

**Figure 4:**
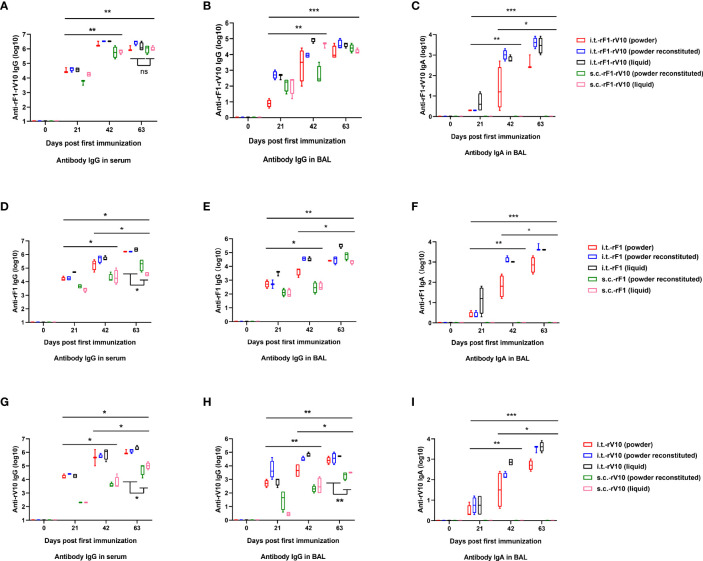
Humoral and mucosal antibody responses (mean ± SD) in mice at 21-, 42- and 63-days post-immunization elicited by rF1-V10, rF1, and rV10 vaccines *via* i.t. (powder, powder reconstituted and liquid formulations) or s.c. (powder reconstituted and liquid formulations). **(A, D, G)** Reciprocal serum titters of IgG to **(A)** rF1-V10, **(D)** rF1, and **(G)** rV10. **(B, E, H)** Reciprocal BAL titers of IgG to **(B)** rF1-V10, **(E)** rF1, and **(H)** rV10. **(C, F, I)** Reciprocal BAL titers of IgA to **(C)** rF1-V10, **(F)** rF1, and **(I)** rV10. Serum and BAL were collected from four mice per group at 21, 42, and 63 dppi and titers were measured by ELISA. Statistical differences were calculated by two-way ANOVA, followed by least significant difference (LSD) analysis or Tukey’s test. *P < 0.05; **P < 0.01; ***P < 0.001.

A substantial and progressive induction of antigen-specific antibody was observed in all groups (**Figure 4**). Booster vaccinations with the same formulation used in the primary vaccine resulted in significant increases in antibody levels at 42 dppi and 63 dppi. At 63 dppi, in rF1-V10-immunized groups, levels of specific IgG in the serum of mice immunized *via* i.t.-rF1-V10 were similar to those for mice immunized *via* s.c.-rF1-V10 (**Figure 4A**). In rF1- and rV10-immunized groups, the levels of IgG in serum of mice immunized *via* i.t.-rF1 or i.t.-rV10 were significantly higher than those in mice immunized *via* s.c.-rF1 or s.c.-rV10 (*P <* 0.05, **Figures 4D, G**). Moreover, no significant differences in levels of specific IgG were observed among different formulations (dry powder, reconstituted powder, liquid) of each subunit vaccine (*P* > 0.05). These results suggest that immunization with rF1-V10 can induce a strong humoral immune response regardless of the delivery route and formulation used.

#### 3.2.2 Lung Mucosal Immune Responses Induced by Vaccines *via* i.t

Since IgG and SIgA both exist in mucosal surfaces (57), the levels of IgG and SIgA in the BAL were tested to evaluate mucosal immune response. The IgG levels in BAL from the i.t.-rF1-V10 and i.t.-rF1 immunized group were comparable to the levels induced in the s.c.-rF1-V10 and s.c.-rF1 immunized group (*P >*0.05), while i.t.-rV10-immunization elicited a significantly higher antibody response than that generated by s.c.-rV10-immunization (*P <*0.05, **Figures 4B, E, H**). The specific SIgA in BAL was not detected in BAL of mice immunized with s.c.-rF1, s.c.-rV10, or s.c.-rF1-V10 (**Figures 4C, F, I**). Antigen-specific SIgA was detected in BAL after immunization with any of the three formulations in i.t.-immunized mice as early as 21 dppi, and levels increased gradually with immunization dose (*P <*0.05, **Figures 4C, F, I**). No significant difference in specific IgA levels in BAL were observed among different vaccine formulations (*P >*0.05). These results indicate that vaccine immunization *via* i.t. effectively induces lung mucosal SIgA production.

### 3.3 Elevated Cellular Immune Response Elicited by rF1, rV10 and rF1-V10 Vaccines *via* i.t

To investigate the specific T cell-mediated immune response, IFN-γ and interleukin (IL)-4 secretion levels were measured. As shown in **Supplementary Figure 1**, the secretion levels of IFN-γ and IL-4 in mice vaccinated *via* i.t. delivery were significantly higher than those in mice vaccinated *via* s.c. delivery (*P <*0.05). No significant difference was observed among different vaccine formulations in i.t.- or s.c.-immunized mice (*P* > 0.05). Vaccine-immunized groups had significantly higher levels of IFN-γ and IL-4 compared to control groups (*P <*0.05).

### 3.4 Enhanced Protection Provided by Immunization With rF1-V10 *via* i.t. or s.c. Following Aerosol Challenge With *Y. pestis* Strain 201

In all immunized groups, the protection conferred by reconstituted powder formulations of vaccines was essentially equal to that conferred by powder or liquid formulations (*P* > 0.05); thus, for clarity, only powder and liquid formulations are shown in the figures.

#### 3.4.1 Protection Efficacy After Aerosol Challenge With 50× LD_50_ of *Y. pestis* Strain 201

**Figure 5:**
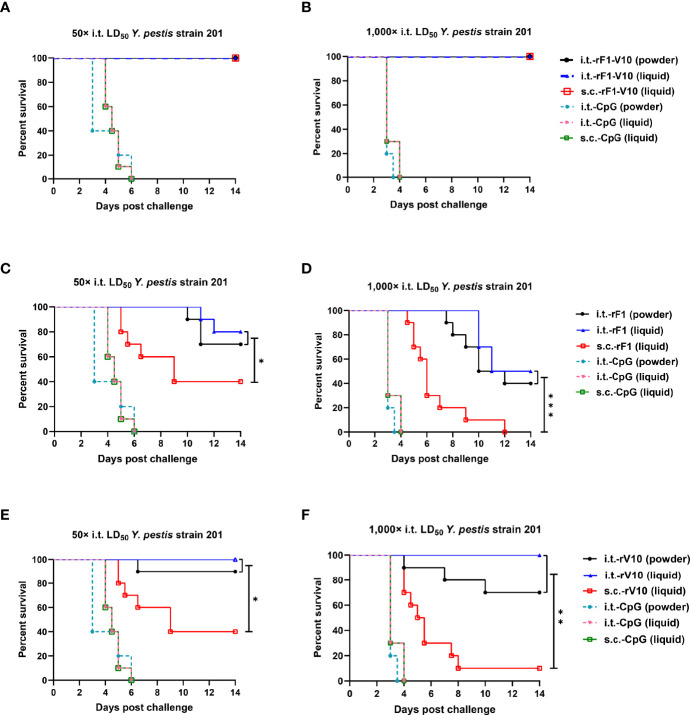
Survival curves for mice challenged with aerosolized *Y. pestis* strain 201. Mice were immunized three times with rF1, rV10, or rF1-V10 in one of three formulations (powder; liquid; or powder reconstituted, not shown) *via* i.t. or s.c. **(A, C, E)** Mice (n = 10) immunized three times with **(A)** rF1-V10, **(C)** rF1, or **(E)** rV10 and then challenged with 50× LD_50_ i.t. at 63 dppi. **(B, D, F)** Mice (n = 10) immunized three times with **(B)** rF1-V10, **(D)** rF1, or **(F)** rV10 and then challenged with 1,000× LD_50_ i.t. For vaccines, inoculations are shown for powder and liquid formulations (powder reconstituted vaccine data are not shown) administered *via* i.t. or s.c. routes. Mortality data were analyzed using Kaplan-Meier survival analysis. *P < 0.05; **P < 0.01; ***P < 0.001.

All mice in PBS (data not shown) and CpG (**Figures 5A, C, E**) control groups died within 6 days. For rF1-V10 immunized groups, immunization, regardless of the formulation and route of administration, conferred 100% protection against lethal challenge doses of *Y. pestis* i.t. (**Figure 5A**). For rF1 immunized groups, the survival rate of mice immunized with powder or liquid (70% or 80% survival, respectively) formulations *via* i.t. was significantly higher than that of mice immunized with liquid (40% survival) formulation *via* s.c. (*P* < 0.05, **Figure 5C**). For rV10 immunized groups, mice immunized with powder or liquid formulation *via* i.t. delivery had significantly higher survival rates (90% and 100% survival, respectively) compared to those immunized with liquid formulation *via* s.c. (40% survival, *P* < 0.05, **Figure 5E**). In addition, the survival of mice receiving s.c.-rF1-V10 immunization (100% survival) was significantly higher than that of mice receiving s.c.-rF1 or s.c.-rV10 immunization (both 40% survival, *P <*0.05, **Figures 5A, C, E**), while the survival rates of mice receiving i.t.-rF1-V10 immunization and those receiving i.t.-rF1 or i.t.-rV10 immunization were not significantly different (*P* > 0.05).

#### 3.4.2 Protection Efficacy After Aerosol Challenge With 1,000× LD_50_ of *Y. pestis* Strain 201

When mice were challenged with a higher dose of 1,000× LD_50_ *Y. pestis* strain 201 i.t. at 63 dppi, all PBS-immunized mice (data not shown) and CpG-immunized mice (**Figures 5B, D, F**) died within 4 days. For rF1-immunized groups, the survival percentage of s.c.-rF1-immunized mice (0% survival) was significantly lower than that of i.t.- rF1-immunized mice (40% survival in powder group; 50% survival in liquid group; *P* < 0.001, **Figure 5D**), and, for each of these groups, survival was lower than when challenged with 50× LD_50_ *Y. pestis* i.t. (40% survival in s.c. groups; 70% survival in i.t.-powder group; 80% survival in i.t.-liquid group). For rV10-immunized groups, survival of s.c.-rV10-immunized mice was further reduced to less than 10%, considerably lower than that of i.t.-rV10-immunized (>70% survival) mice (*P* < 0.01, **Figure 5F**). However, i.t.-rV10 immunization with liquid formulation and rF1-V10 immunization (all formulations and routes) still conferred complete protection against 1,000× LD_50_ i.t. *Y. pestis* challenge. Overall, these results indicate that rF1-V10 is an excellent subunit vaccine and that the i.t. immunization route is superior to the s.c. immunization route for protection against *Y. pestis* i.t. infection.

### 3.5 Bacterial Enumeration From Mouse Organs Following i.t. Challenge

**Figure 6:**
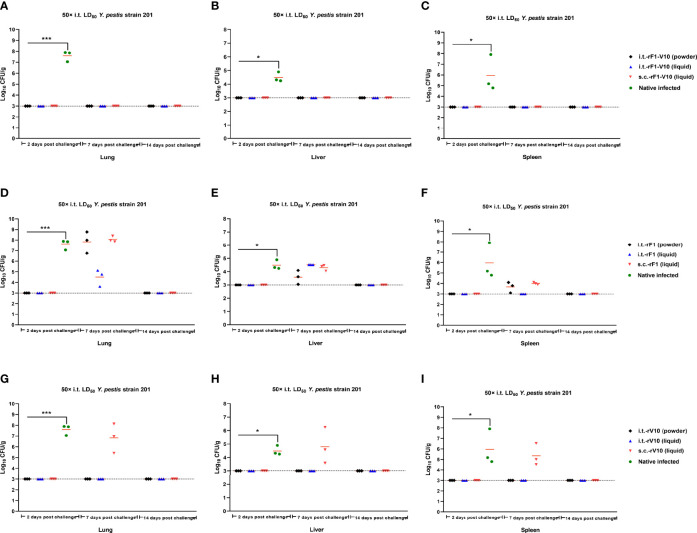
Bacterial load in the organs of mice euthanized at days 2, 7, and 14 post 50× LD_50_ of *Y. pestis* strain 201 i.t. challenge. **(A–C)** Bacterial load in **(A)** lungs, **(B)** liver, and **(C)** spleen of mice immunized with rF1-V10 dry power and liquid formulations. **(D–F)** Bacterial loads in **(D)** lungs, **(E)** liver and **(F)** spleen of mice immunized with rF1 dry power and liquid formulations. **(G–I)** Bacterial loads in **(G)** lungs, **(H)** liver and **(I)** spleen of mice immunized with rV10 dry power and liquid formulations. Experiments were performed twice independently with similar results. Data are expressed as the mean ± SD (n = 3) using data collected from one representative experiment. *P <0.05; ***P <0.001.

Bacterial loads in different tissues of vaccine-immunized mice following i.t challenge at 50× LD_50_ *Y. pestis* were examined to further evaluate the protection provided by subunit vaccine immunization. At day 2 post-challenge, bacterial loads were below the lowest detectable limit of the assay for lungs, livers, and spleens from i.t.-rF1, s.c.-rF1 and s.c.-rV10-immunized groups; at day 7 post-challenge, bacterial loads ranged from 10^3^ CFU/g to 10^7^ CFU/g, indicating a mild proliferation of *Y. pestis* in these groups. But by day 14 post-challenge, bacteria had been cleared from tissues (**Figures 6D–I**). No bacteria were detected at the lowest dilution of organs from rF1-V10-immunized mice at days 2, 7 or 14 post-challenges (**Figures 6A–C**), indicating that immunization with rF1-V10, regardless of route or formulation, had an appreciable protective effect against *Y. pestis*. For control groups, extensive bacteria were found in lungs, spleens, and livers of naive mice at day 2 post-challenge, and all naive mice succumbed to the *Y. pestis* challenge by day 6 post-challenge.

### 3.6 Histopathological Analysis of Mouse Tissues After Immunization and Challenge

The safety of i.t. vaccine immunization was evaluated by examining pathological changes in mouse tissues after vaccination. No obvious pathological lesions were observed in lungs, livers, or spleens of mice immunized with any formulation of vaccines, with CpG, or with PBS *via* i.t. or s.c. routes, confirming the safety of i.t. immunization (**Supplementary Figures 2A–C**).

**Figure 7:**
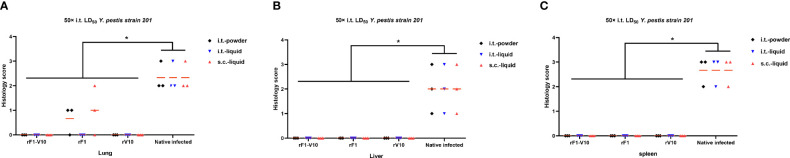
Pathological lesions in the tissues of mice euthanized at 2 days post 50× LD_50_ *Y. pestis* strain 201 i.t. challenge. Tissue from lungs, livers, and spleens were collected from three mice per group (rF1-V10, rF1, and rV10 with different formulations *via* i.t. or s.c. routes), fixed in formalin, embedded in paraffin, and stained with HE. Pathological scores of **(A)** lung tissue; **(B)** liver tissue; and **(C)** spleen tissue are presented. Tissue sections were evaluated by a trained pathologist according to the following scores: 0, no pathological lesions; 1, minimal; 2, mild; 3, moderate; 4, severe. The degree of pathological lesions was related to the distribution and severity of lesions as follows: (I) edema; (II) tissue parenchymatous lesions, such as congestion and hemorrhage. Experiments were performed twice independently with similar results. Data are expressed as the mean ± SD (n = 3) for data collected from one representative experiment. *P < 0.05.

The efficacy of different vaccines was further assessed by comparing the pathological changes in mouse tissues after 50× LD_50_ i.t. challenge. In unvaccinated infected mice, perivascular edema, neutrophil infiltration, and hemorrhage were found in the lungs, and inflammatory cells were recruited to the liver and the white pulp of the spleen (**Supplementary Figures 2D–F**). No obvious lesions were observed in vaccine-immunized groups, except for the rF1-immunized groups, which had mild inflammation in the lungs. Furthermore, the pathological scores in the lungs, livers, and spleens of the unvaccinated infected control group were significantly higher than those of the vaccine-immunized groups (*P <*0.05, **Figure 7**).

## 4 Discussion

Due to the sporadic outbreak of pneumonic plague and the threat should it be weaponized, developing effective vaccines against pneumonic plague is important. Compared with F1 or LcrV alone, a subunit vaccine based on a fusion of F1-LcrV proteins is a promising subunit vaccine (30, 31). Previous studies reported that immunization with the rF1-LcrV fusion protein through the s.c. route could fully protect mice only at 10× LD_50_ virulent *Y. pestis* CO92 *via* intranasal (i.n.) challenge (34) and only partially protect mice at 70× LD_50_ *via* inhalational challenge (58). The injectable vaccine also failed to adequately protect African green monkeys against aerosolized *Y. pestis* (19). Other reports have shown that rF1-LcrV immunization through the non-invasive i.n. route conferred 80-90% protection against 70 to 100× LD_50_ of *Y. pestis via* inhalational challenge (58, 59). Finally, Jones et al. (32) demonstrated that immunization with Protollin-F1-V through an i.n. route elicits 80% protection against an aerosol challenge at 255× LD_50_ of *Y. pestis* and 100% protection against 170× LD_50_ of *Y. pestis*. Overall, these studies indicate that the route of inoculation affects vaccine effectiveness against pneumonic plague. Modifying the antigen, we created the rF1-V10 fusion protein and established a mouse model of i.t. delivery to investigate protective immunity against pneumonic plague. Our results demonstrate that i.t.-rF1-V10 immunization induces strong immune responses that lead to 100% protection against an aerosol challenge of high-dose *Y. pestis* strain 201. Moreover, i.t. inoculation is an improved non-invasive method for pulmonary delivery of vaccines in mice compared to conventional i.n. inoculation that has several disadvantages: a low residence time; mucociliary clearance of vaccines in the nose, throat and upper airways leads to an inefficient uptake of soluble antigens; the route is unsuitable for powder pulmonary delivery; and it is not possible to quantify the given dose (60–62). We propose that the i.t. immunization with inhalable rF1-V10 vaccine may provide an alternative, possibly better, vaccination strategy against pneumonic plague.

Compared to pulmonary delivery (i.n., i.t. etc.) of liquid vaccine, powder formulation delivered by i.t. route is more advantageous (63). The F1-V SFD powders reconstituted in water delivered by i.n. route provided at most 80% protection against bubonic plague, however, the immunization of powder formulation was not included in this previous study (64). We recently demonstrated the liquid formulation of EV76-B-SHUΔ*pla* delivered by i.t. route represents an excellent live-attenuated vaccine candidate against pneumonic plague (54), but it is likely unsuitable for preparation of powder by SFD, which will affect survivability and overall fitness of the live bacteria. In the current study, we evaluated the protection efficacy of three subunit vaccines administrated with three different formulations *via* i.t. route. Our results demonstrate that protection against an aerosolized *Y. pestis* challenge conferred by i.t. delivery of vaccines in dry powder formulations is at least equivalent to that conferred by i.t. delivery of liquid formulations of the same vaccine. Potential advantages of dry powder over liquid vaccines include: (i) the powder formulations could eliminate the cold-chain requirement, thus considerably reducing the costs of storage and shipping (60); (ii) the improved antigen stability may enhance the immunity induced after vaccination (65); (iii) using excipients as bulking agent could increase the total amount of inhaled powders, which makes given dose more accurate (66). These advantages suggest that powder formulation immunization *via* i.t. can provide a promising improvement over the existing vaccine.

At the mucosal surfaces, the predominant immunoglobulin is secretory IgA (SIgA). SIgA-based protective mucosal immunity can prevent an infectious agent from entering the body and block microbial toxins from binding to, or affecting, epithelial and other target cells (67). Some studies suggest that specific SIgA plays a key role in neutralizing pathogens or toxins (68, 69). One limitation of this study is that we didn’t explain why s.c.-rF1-V10 vaccination conferred complete protection against a high dose *Y. pestis* challenge without inducing SIgA production. This result indicated that the role of specific SIgA in protecting against respiratory infection of *Y. pestis* needs to be further investigated, such as evaluating the survival rate of mice challenged with *Y. pestis* that had been preincubated with SIgA (70), or evaluating the efficacy of subunit vaccines *via* i.t. delivery in wild-type (WT) and IgA-deficient (IgA (-/-)) mice (71). Another limitation is that we didn’t evaluate the innate immune response in the early stage of i.t. immunization, which is supposed to be more efficient than that of s.c. immunization (72, 73).

Taken together, we have demonstrated that the rF1-V10 fusion protein vaccine can confer complete protection against a high dose aerosolized *Y. pestis* challenge and that i.t. delivery of vaccines can induce higher protection efficacy in mice compared to that of s.c. immunization. However, i.t. route might be only suitable for animal use. For human pulmonary delivery of dry powder vaccine, the widely accepted inhaled devices named dry powder inhalers (DPIs) are recommended, which are easy to administrate without the assistant of trained medical personnel (65, 74). The use of this alternative method of pulmonary delivery and powder vaccine formulations may directly benefit biodefense vaccination programs and, ultimately, facilitate mass vaccination.

## Data Availability Statement

The datasets presented in this study can be found in online repositories. The names of the repository/repositories and accession number(s) can be found in the article/**Supplementary Material**.

## Ethics Statement

The animal study was reviewed and approved by the Institute of Animal Care and Use Committee (IACUC) at the Academy of Military Medical Sciences (AMMS).

## Author Contributions

DZ, XX, and WY designed the experiments. WZ, XS, LLZ, and LNZ performed animal experiments. ML and LH finished other experiments together. JG and XZ analyzed data and drew the figures. WZ, XS, DZ, XX, and WY revised the manuscript. All authors approved the article.

## Conflict of Interest

The authors declare that the research was conducted in the absence of any commercial or financial relationships that could be construed as a potential conflict of interest.

## Publisher’s Note

All claims expressed in this article are solely those of the authors and do not necessarily represent those of their affiliated organizations, or those of the publisher, the editors and the reviewers. Any product that may be evaluated in this article, or claim that may be made by its manufacturer, is not guaranteed or endorsed by the publisher.

## References

[1] Feodorova VA Corbel MJ. Prospects for New Plague Vaccines. Expert Rev Vaccines (2009) 8(12):1721–38. doi: 10.1586/erv.09.129 19943765

[2] Drancourt M Raoult D. Molecular History of Plague. Clin Microbiol Infect (2016) 22(11):911–5. doi: 10.1016/j.cmi.2016.08.031 27615720

[3] Ke Y Chen Z Yang R. Yersinia Pestis: Mechanisms of Entry Into and Resistance to the Host Cell. Front Cell Infect Microbiol (2013) 3:106. doi: 10.3389/fcimb.2013.00106 24400226 PMC3871965

[4] Verma SK Tuteja U. Plague Vaccine Development: Current Research and Future Trends. Front Immunol (2016) 7:602. doi: 10.3389/fimmu.2016.00602 28018363 PMC5155008

[5] Barbieri R Signoli M Chevé D Costedoat C Tzortzis S Aboudharam G. Yersinia Pestis: The Natural History of Plague. Clin Microbiol Rev (2020) 34(1):e00044–19. doi: 10.1128/cmr.00044-19 PMC7920731 33298527

[6] Perry RD Fetherston JD. Yersinia Pestis–Etiologic Agent of Plague. Clin Microbiol Rev (1997) 10(1):35–66. doi: 10.1128/cmr.10.1.35 8993858 PMC172914

[7] Yang R. Plague: Recognition, Treatment, and Prevention. J Clin Microbiol (2018) 56(1):e01519–17. doi: 10.1128/jcm.01519-17 PMC5744195 29070654

[8] Sebbane F Jarrett CO Gardner D Long D Hinnebusch BJ. Role of the Yersinia Pestis Plasminogen Activator in the Incidence of Distinct Septicemic and Bubonic Forms of Flea-Borne Plague. Proc Natl Acad Sci U S A (2006) 103(14):5526–30. doi: 10.1073/pnas.0509544103 PMC1414629 16567636

[9] Pechous RD Sivaraman V Stasulli NM Goldman WE. Pneumonic Plague: The Darker Side of Yersinia Pestis. Trends Microbiol (2016) 24(3):190–7. doi: 10.1016/j.tim.2015.11.008 26698952

[10] Prentice MB Rahalison L. Plague. Lancet (2007) 369(9568):1196–207. doi: 10.1016/s0140-6736(07)60566-2 17416264

[11] Inglesby TV Dennis DT Henderson DA Bartlett JG Ascher MS Eitzen E. Plague as a Biological Weapon: Medical and Public Health Management. Working Group on Civilian Biodefense. Jama (2000) 283(17):2281–90. doi: 10.1001/jama.283.17.2281 10807389

[12] Meyer KF. Effectiveness of Live or Killed Plague Vaccines in Man. Bull World Health Organ (1970) 42(5):653–66. PMC2427500 4988692

[13] Russell P Eley SM Hibbs SE Manchee RJ Stagg AJ Titball RW. A Comparison of Plague Vaccine, USP and EV76 Vaccine Induced Protection Against Yersinia Pestis in a Murine Model. Vaccine (1995) 13(16):1551–6. doi: 10.1016/0264-410x(95)00090-n 8578841

[14] Cohen RJ Stockard JL. Pneumonic Plague in an Untreated Plague-Vaccinated Individual. Jama (1967) 202(4):365–6. doi: 10.1001/jama.1967.03130170165036 6072311

[15] Titball RW Williamson ED. Yersinia Pestis (Plague) Vaccines. Expert Opin Biol Ther (2004) 4(6):965–73. doi: 10.1517/14712598.4.6.965 15174978

[16] Girard G. [Immunity in Plague. Acquisitions Supplied by 30 Years of Work on The "Pasteurella Pestis Ev" (Girard and Robic) Strain]. Biol Med (1963) 52:631–731. L'immunit'e dans l'infection pesteuse. Acquisitions apport'ees par 30 ann'ees de travaux sur la souche de "pasteurella pestis ev" (girard et robic). fre. 14095162

[17] Wang X Zhang X Zhou D Yang R. Live-Attenuated Yersinia Pestis Vaccines. Expert Rev Vaccines (2013) 12(6):677–86. doi: 10.1586/erv.13.42 23750796

[18] Feodorova VA Sayapina LV Corbel MJ Motin VL. Russian Vaccines Against Especially Dangerous Bacterial Pathogens. Emerg Microbes Infect (2014) 3(12):e86. doi: 10.1038/emi.2014.82 26038506 PMC4317636

[19] Smiley ST. Current Challenges in the Development of Vaccines for Pneumonic Plague. Expert Rev Vaccines (2008) 7(2):209–21. doi: 10.1586/14760584.7.2.209 PMC2365752 18324890

[20] Baker EE Sommer H Foster LE Meyer E Meyer KF. Studies on Immunization Against Plague. I. The Isolation and Characterization of the Soluble Antigen of Pasteurella Pestis. J Immunol (1952) 68(2):131–45. 14927919

[21] Andrews GP Heath DG Anderson GW Jr Welkos SL Friedlander AM. Fraction 1 Capsular Antigen (F1) Purification From Yersinia Pestis CO92 and From an Escherichia Coli Recombinant Strain and Efficacy Against Lethal Plague Challenge. Infect Immun (1996) 64(6):2180–7. doi: 10.1128/iai.64.6.2180-2187.1996 PMC174053 8675324

[22] Sha J Endsley JJ Kirtley ML Foltz SM Huante MB Erova TE. Characterization of an F1 Deletion Mutant of Yersinia Pestis CO92, Pathogenic Role of F1 Antigen in Bubonic and Pneumonic Plague, and Evaluation of Sensitivity and Specificity of F1 Antigen Capture-Based Dipsticks. J Clin Microbiol (2011) 49(5):1708–15. doi: 10.1128/jcm.00064-11 PMC3122665 21367990

[23] Burrows TW. An Antigen Determining Virulence in Pasteurella Pestis. Nature (1956) 177(4505):426–7. doi: 10.1038/177426b0 13309325

[24] Anderson GW Jr Leary SE Williamson ED Titball RW Welkos SL Worsham PL. Recombinant V Antigen Protects Mice Against Pneumonic and Bubonic Plague Caused by F1-Capsule-Positive and -Negative Strains of Yersinia Pestis. Infect Immun (1996) 64(11):4580–5. doi: 10.1128/iai.64.11.4580-4585.1996 PMC174416 8890210

[25] Batra L Verma SK Nagar DP Saxena N Pathak P Pant SC. HSP70 Domain II of Mycobacterium Tuberculosis Modulates Immune Response and Protective Potential of F1 and LcrV Antigens of Yersinia Pestis in a Mouse Model. PloS Negl Trop Dis (2014) 8(12):e3322. doi: 10.1371/journal.pntd.0003322 25474358 PMC4256173

[26] Nakajima R Motin VL Brubaker RR. Suppression of Cytokines in Mice by Protein A-V Antigen Fusion Peptide and Restoration of Synthesis by Active Immunization. Infect Immun (1995) 63(8):3021–9. doi: 10.1128/iai.63.8.3021-3029.1995 PMC173411 7622225

[27] Overheim KA Depaolo RW Debord KL Morrin EM Anderson DM Green NM. LcrV Plague Vaccine With Altered Immunomodulatory Properties. Infect Immun (2005) 73(8):5152–9. doi: 10.1128/iai.73.8.5152-5159.2005 PMC1201268 16041032

[28] Liu L Wei D Qu Z Sun L Miao Y Yang Y. A Safety and Immunogenicity Study of a Novel Subunit Plague Vaccine in Cynomolgus Macaques. J Appl Toxicol (2018) 38(3):408–17. doi: 10.1002/jat.3550 29134676

[29] Qi Z Zhou L Zhang Q Ren L Dai R Wu B. Comparison of Mouse, Guinea Pig and Rabbit Models for Evaluation of Plague Subunit Vaccine F1+Rv270. Vaccine (2010) 28(6):1655–60. doi: 10.1016/j.vaccine.2009.02.078 20079562

[30] Williamson ED Eley SM Griffin KF Green M Russell P Leary SE. A New Improved Sub-Unit Vaccine for Plague: The Basis of Protection. FEMS Immunol Med Microbiol (1995) 12(3-4):223–30. doi: 10.1111/j.1574-695X.1995.tb00196.x 8745007

[31] Williamson ED Sharp GJ Eley SM Vesey PM Pepper TC Titball RW. Local and Systemic Immune Response to a Microencapsulated Sub-Unit Vaccine for Plague. Vaccine (1996) 14(17-18):1613–9. doi: 10.1016/s0264-410x(96)00151-x 9032889

[32] Jones T Adamovicz JJ Cyr SL Bolt CR Bellerose N Pitt LM. Intranasal Protollin/F1-V Vaccine Elicits Respiratory and Serum Antibody Responses and Protects Mice Against Lethal Aerosolized Plague Infection. Vaccine (2006) 24(10):1625–32. doi: 10.1016/j.vaccine.2005.09.052 16243411

[33] Erova TE Rosenzweig JA Sha J Suarez G Sierra JC Kirtley ML. Evaluation of Protective Potential of Yersinia Pestis Outer Membrane Protein Antigens as Possible Candidates for a New-Generation Recombinant Plague Vaccine. Clin Vaccine Immunol (2013) 20(2):227–38. doi: 10.1128/cvi.00597-12 PMC3571283 23239803

[34] Bowen W Batra L Pulsifer AR Yolcu ES Lawrenz MB Shirwan H. Robust Th1 Cellular and Humoral Responses Generated by the Yersinia Pestis Rf1-V Subunit Vaccine Formulated to Contain an Agonist of the CD137 Pathway do Not Translate Into Increased Protection Against Pneumonic Plague. Vaccine (2019) 37(38):5708–16. doi: 10.1016/j.vaccine.2019.07.103 PMC6773249 31416643

[35] Feodorova VA Motin VL. Plague Vaccines: Current Developments and Future Perspectives. Emerg Microbes Infect (2012) 1(11):e36. doi: 10.1038/emi.2012.34 26038406 PMC3630923

[36] Lyadova IV Vordermeier HM Eruslanov EB Khaidukov SV Apt AS Hewinson RG. Intranasal BCG Vaccination Protects BALB/c Mice Against Virulent Mycobacterium Bovis and Accelerates Production of IFN-Gamma in Their Lungs. Clin Exp Immunol (2001) 126(2):274–9. doi: 10.1046/j.1365-2249.2001.01667.x PMC1906185 11703371

[37] Garcia-Contreras L Wong YL Muttil P Padilla D Sadoff J Derousse J. Immunization by a Bacterial Aerosol. Proc Natl Acad Sci U S A (2008) 105(12):4656–60. doi: 10.1073/pnas.0800043105 PMC2290758 18344320

[38] Lycke N. Recent Progress in Mucosal Vaccine Development: Potential and Limitations. Nat Rev Immunol (2012) 12(8):592–605. doi: 10.1038/nri3251 22828912

[39] Patton JS Byron PR. Inhaling Medicines: Delivering Drugs to the Body Through the Lungs. Nat Rev Drug Discov (2007) 6(1):67–74. doi: 10.1038/nrd2153 17195033

[40] Bramwell VW Eyles JE Oya Alpar H. Particulate Delivery Systems for Biodefense Subunit Vaccines. Adv Drug Deliv Rev (2005) 57(9):1247–65. doi: 10.1016/j.addr.2005.01.010 15935873

[41] Illum L Jabbal-Gill I Hinchcliffe M Fisher AN Davis SS. Chitosan as a Novel Nasal Delivery System for Vaccines. Adv Drug Deliv Rev (2001) 51(1-3):81–96. doi: 10.1016/s0169-409x(01)00171-5 11516781

[42] GarmiseRJStaatsHFHickeyAJ. Novel Dry Powder Preparations of Whole Inactivated Influenza Virus for Nasal Vaccination. AAPS PharmSciTech (2007) 8(4):E81. doi: 10.1208/pt0804081 18181542

[43] Coulanges P. [50th Anniversary of the EV Antiplague Vaccine (Girard and Robic)]. Bull Soc Pathol Exot Filiales (1983) 76(2):114–20. Cinquantenaire du vaccin antipesteux EV (Girard et Robic). fre. 6347412

[44] Coulanges P. [The 50th Anniversary of the Anti-Plague Vaccine EV (Girard and Robic)]. Arch Inst Pasteur Madagascar (1982) 50(1):169–84. Cinquantenaire du vaccin antipesteux EV (Girard et Robic). fre. 6764608

[45] Meyer KF Smith G Foster L Brookman M Sung M. Live, Attenuated Yersinia Pestis Vaccine: Virulent in Nonhuman Primates, Harmless to Guinea Pigs. J Infect Dis (1974) 129:Suppl:S85–12. doi: 10.1093/infdis/129.supplement_1.s85 4207627

[46] Powell BS Andrews GP Enama JT Jendrek S Bolt C Worsham P. Design and Testing for a Nontagged F1-V Fusion Protein as Vaccine Antigen Against Bubonic and Pneumonic Plague. Biotechnol Prog (2005) 21(5):1490–510. doi: 10.1021/bp050098r 16209555

[47] Sing A Rost D Tvardovskaia N Roggenkamp A Wiedemann A Kirschning CJ. Yersinia V-Antigen Exploits Toll-Like Receptor 2 and CD14 for Interleukin 10-Mediated Immunosuppression. J Exp Med (2002) 196(8):1017–24. doi: 10.1084/jem.20020908 PMC2194041 12391013

[48] Sawa T Yahr TL Ohara M Kurahashi K Gropper MA Wiener-Kronish JP. Active and Passive Immunization With the Pseudomonas V Antigen Protects Against Type III Intoxication and Lung Injury. Nat Med (1999) 5(4):392–8. doi: 10.1038/7391 10202927

[49] Torge A Grützmacher P Mücklich F Schneider M. The Influence of Mannitol on Morphology and Disintegration of Spray-Dried Nano-Embedded Microparticles. Eur J Pharm Sci (2017) 104:171–9. doi: 10.1016/j.ejps.2017.04.003 28390837

[50] Gan C Luo W Yu Y Jiao Z Li S Su D. Intratracheal Inoculation of AHc Vaccine Induces Protection Against Aerosolized Botulinum Neurotoxin A Challenge in Mice. NPJ Vaccines (2021) 6(1):87. doi: 10.1038/s41541-021-00349-w 34158496 PMC8219734

[51] Fukuyama Y Ikeda Y Ohori J Sugita G Aso K Fujihashi K. A Molecular Mucosal Adjuvant to Enhance Immunity Against Pneumococcal Infection in the Elderly. Immune Netw (2015) 15(1):9–15. doi: 10.4110/in.2015.15.1.9 25713504 PMC4338268

[52] HarrisJASteinSWMyrdalPB. Evaluation of the TSI Aerosol Impactor 3306/3321 System Using a Redesigned Impactor Stage With Solution and Suspension Metered-Dose Inhalers. AAPS PharmSciTech (2006) 7(1):E138–e145. doi: 10.1208/pt070120 28290035

[53] Tian G Qi Z Qiu Y Wu X Zhang Q Yang X. Comparison of Virulence Between the Yersinia Pestis Microtus 201, an Avirulent Strain to Humans, and the Vaccine Strain EV in Rhesus Macaques, Macaca Mulatta. Hum Vaccin Immunother (2014) 10(12):3552–60. doi: 10.4161/hv.35119 PMC4514055 25483697

[54] Feng J Deng Y Fu M Hu X Luo W Lu Z. Construction of a Live-Attenuated Vaccine Strain of Yersinia Pestis EV76-B-ShuΔpla and Evaluation of Its Protection Efficacy in a Mouse Model by Aerosolized Intratracheal Inoculation. Front Cell Infect Microbiol (2020) 10:473. doi: 10.3389/fcimb.2020.00473 33014895 PMC7509399

[55] Feng J Hu X Fu M Dai L Yu Y Luo W. Enhanced Protection Against Q Fever in BALB/c Mice Elicited by Immunization of Chloroform-Methanol Residue of Coxiella Burnetii *via* Intratracheal Inoculation. Vaccine (2019) 37(41):6076–84. doi: 10.1016/j.vaccine.2019.08.041 31477436

[56] Xiong X Qi Y Jiao J Gong W Duan C Wen B. Exploratory Study on Th1 Epitope-Induced Protective Immunity Against Coxiella Burnetii Infection. PloS One (2014) 9(1):e87206. doi: 10.1371/journal.pone.0087206 24498044 PMC3907486

[57] Chen K Magri G Grasset EK Cerutti A. Rethinking Mucosal Antibody Responses: IgM, IgG and IgD Join IgA. Nat Rev Immunol (2020) 20(7):427–41. doi: 10.1038/s41577-019-0261-1 PMC10262260 32015473

[58] Glynn A Roy CJ Powell BS Adamovicz JJ Freytag LC Clements JD. Protection Against Aerosolized Yersinia Pestis Challenge Following Homologous and Heterologous Prime-Boost With Recombinant Plague Antigens. Infect Immun (2005) 73(8):5256–61. doi: 10.1128/iai.73.8.5256-5261.2005 PMC1201190 16041052

[59] Yamanaka H Hoyt T Yang X Golden S Bosio CM Crist K. A Nasal Interleukin-12 DNA Vaccine Coexpressing Yersinia Pestis F1-V Fusion Protein Confers Protection Against Pneumonic Plague. Infect Immun (2008) 76(10):4564–73. doi: 10.1128/iai.00581-08 PMC2546814 18694965

[60] Amorij JP Saluja V Petersen AH Hinrichs WL Huckriede A Frijlink HW. Pulmonary Delivery of an Inulin-Stabilized Influenza Subunit Vaccine Prepared by Spray-Freeze Drying Induces Systemic, Mucosal Humoral as Well as Cell-Mediated Immune Responses in BALB/c Mice. Vaccine (2007) 25(52):8707–17. doi: 10.1016/j.vaccine.2007.10.035 17996993

[61] Jabbal-Gill I. Nasal Vaccine Innovation. J Drug Target (2010) 18(10):771–86. doi: 10.3109/1061186x.2010.523790 21047271

[62] Tomar J Born PA Frijlink HW Hinrichs WL. Dry Influenza Vaccines: Towards a Stable, Effective and Convenient Alternative to Conventional Parenteral Influenza Vaccination. Expert Rev Vaccines (2016) 15(11):1431–47. doi: 10.1080/14760584.2016.1182869 27118428

[63] Beck SE Laube BL Barberena CI Fischer AC Adams RJ Chesnut K. Deposition and Expression of Aerosolized rAAV Vectors in the Lungs of Rhesus Macaques. Mol Ther J Am Soc Gene Ther (2002) 6(4):546–54. doi: 10.1006/mthe.2002.0698 12387250

[64] Huang J D'Souza AJ Alarcon JB Mikszta JA Ford BM Ferriter MS. Protective Immunity in Mice Achieved With Dry Powder Formulation and Alternative Delivery of Plague F1-V Vaccine. Clin Vaccine Immunol (2009) 16(5):719–25. doi: 10.1128/cvi.00447-08 PMC2681576 19261773

[65] Sou T Meeusen EN de Veer M Morton DA Kaminskas LM McIntosh MP. New Developments in Dry Powder Pulmonary Vaccine Delivery. Trends Biotechnol (2011) 29(4):191–8. doi: 10.1016/j.tibtech.2010.12.009 21255854

[66] Tonnis WF Lexmond AJ Frijlink HW de Boer AH Hinrichs WL. Devices and Formulations for Pulmonary Vaccination. Expert Opin Drug Deliv (2013) 10(10):1383–97. doi: 10.1517/17425247.2013.810622 23786408

[67] Mantis NJ Rol N Corthésy B. Secretory IgA's Complex Roles in Immunity and Mucosal Homeostasis in the Gut. Mucosal Immunol (2011) 4(6):603–11. doi: 10.1038/mi.2011.41 PMC3774538 21975936

[68] Holmgren J Czerkinsky C. Mucosal Immunity and Vaccines. Nat Med (2005) 11(4 Suppl):S45–53. doi: 10.1038/nm1213 15812489

[69] Mantis NJ McGuinness CR Sonuyi O Edwards G Farrant SA. Immunoglobulin A Antibodies Against Ricin A and B Subunits Protect Epithelial Cells From Ricin Intoxication. Infect Immun (2006) 74(6):3455–62. doi: 10.1128/iai.02088-05 PMC1479255 16714576

[70] Kobayashi R Kohda T Kataoka K Ihara H Kozaki S Pascual DW. A Novel Neurotoxoid Vaccine Prevents Mucosal Botulism. J Immunol (2005) 174(4):2190–5. doi: 10.4049/jimmunol.174.4.2190 15699151

[71] Rodríguez A Tjärnlund A Ivanji J Singh M García I Williams A. Role of IgA in the Defense Against Respiratory Infections IgA Deficient Mice Exhibited Increased Susceptibility to Intranasal Infection With Mycobacterium Bovis BCG. Vaccine (2005) 23(20):2565–72. doi: 10.1016/j.vaccine.2004.11.032 15780438

[72] Piggott DA Eisenbarth SC Xu L Constant SL Huleatt JW Herrick CA. MyD88-Dependent Induction of Allergic Th2 Responses to Intranasal Antigen. J Clin Invest (2005) 115(2):459–67. doi: 10.1172/jci22462 PMC544038 15650773

[73] Neutra MR Kozlowski PA. Mucosal Vaccines: The Promise and the Challenge. Nat Rev Immunol (2006) 6(2):148–58. doi: 10.1038/nri1777 16491139

[74] Dal Negro RW. Dry Powder Inhalers and the Right Things to Remember: A Concept Review. Multidiscip Respir Med (2015) 10(1):13. doi: 10.1186/s40248-015-0012-5 25878791 PMC4397837

