# Supplementary material for: Complete Protection Against Yersinia pestis in BALB/c Mouse Model Elicited by Immunization With Inhalable Formulations of rF1-V10 Fusion Protein via Aerosolized Intratracheal Inoculation
Source: Front Immunol. 2022 Jan 26;13:793382. doi: 10.3389/fimmu.2022.793382 (PMC8825376; doi:10.3389/fimmu.2022.793382)
Supplement: Supplementary file 1 [file DataSheet_1.pdf]

Supplementary Material

1 Supplementary Figures and Tables

1.1 Supplementary Tables

SUPPLEMENTARY TABLE 1. List of primers and their sequences used for construction of rF1, rV10, and rF1-rV10

Primer	Sequence (5'-3')
rF1-F0	***GGCTCACAGAGAACAGATTGGTGGC***ATGGCAGATTTAACTGCAAGC
rF1-R0	***AGCCGGATCTCAGTGGTGGTGGTGGTGGTGCTCGAG***TTATTGGTTAGATACGGTTACG
rV10-F0	***GGCTCACAGAGAACAGATTGGTGGC***ATGATTAGAGCCTACGAACAAAAC
rV10-R0	***AGCCGGATCTCAGTGGTGGTGGTGGTGGTGCTCGAG***TCATTTACCAGACGTGTCATCT
rV10-F-linker	CTTTGCC***GGCACC***GCTATTGAAGCACTGAACCG
rV10-R-linker	CAATAGC***GGTGCC***GGCAAAGTGAGATAATTCATT
rF1-V10-F-linker	CTAACCAA***GAATTT***ATTAGAGCCTACGAACAAAAC
rF1-V10-R-linker	CTCTAAT***AAATTC***TTGGTTAGATACGGTTACGG

Bold and italicized letters represent the DNA sequence encoding the linker.
*Bold, italicized, and underlined letters represent the DNA sequence encoding the homology arm for the building of recombinant plasmids.

1.2 Supplementary Figures

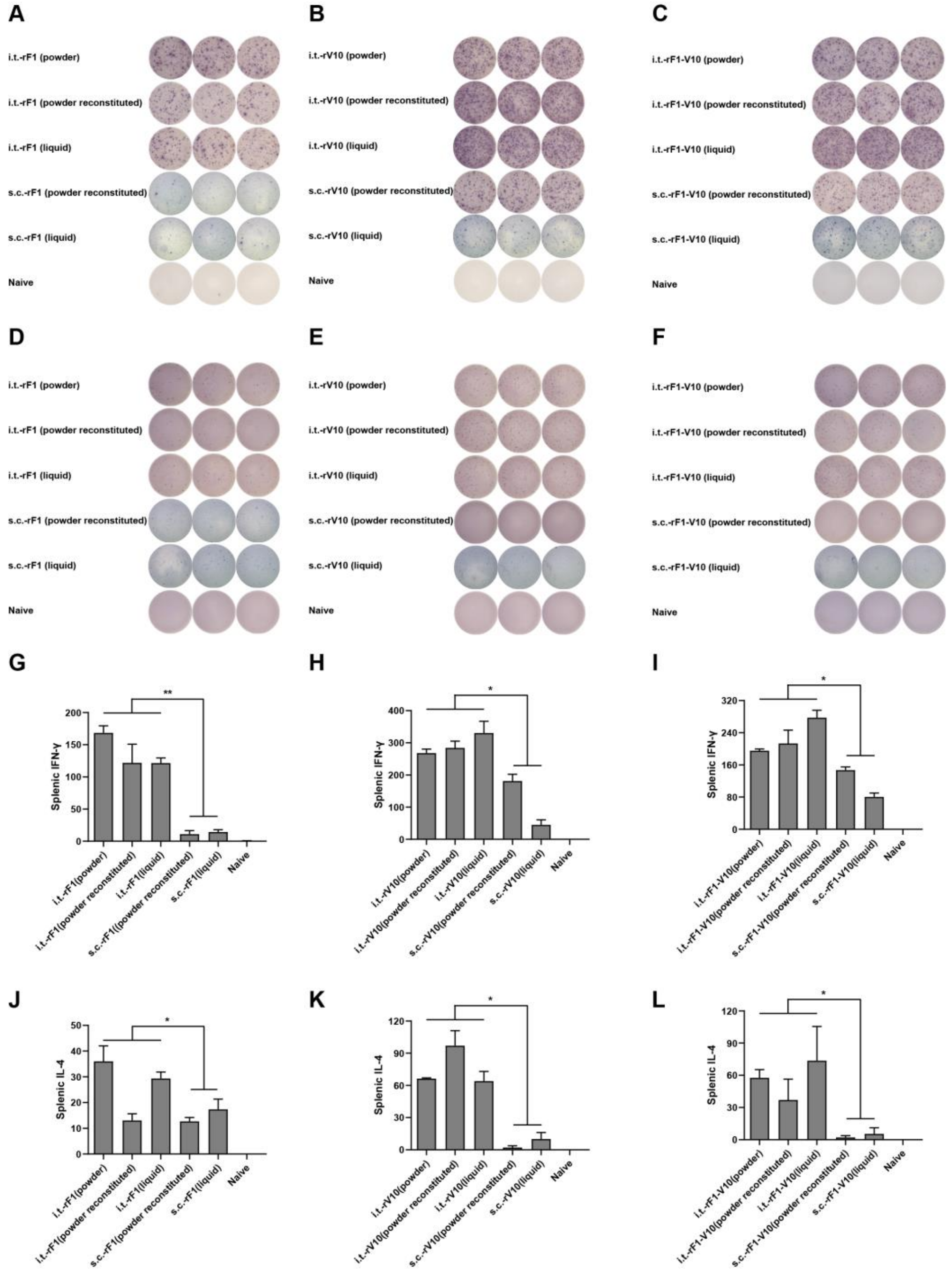

Supplementary Figure 1. IFN-γ and IL-4 ELISPOT-based quantification of antigen-specific T cells in mice. At 63 dppi, T cells were isolated from the spleens of four mice in each group and stimulated with cognate rF1, rV10, or rF1-V10 for 20 h. **(A-C, G-I)** Quantification of antigen-specific IFN-γ-producing T cells. **(D-F, J-L)** Quantification of antigen-specific IL-4-producing T cells. **(A, D, G, J)** rF1 immunization with three formulations via i.t. or s.c. **(B, E, H, K)** rV10 immunization with three formulations via i.t. or s.c. **(C, F, I, L)** rF1-V10 immunization with three formulations via i.t. or s.c. The levels of IFN-γ and IL-4 were analyzed using an Biosys Bioreader 7000. Data are expressed as mean ± SD (n = 4). $^{**}P < 0.01$; $^{*}P < 0.05$.

A

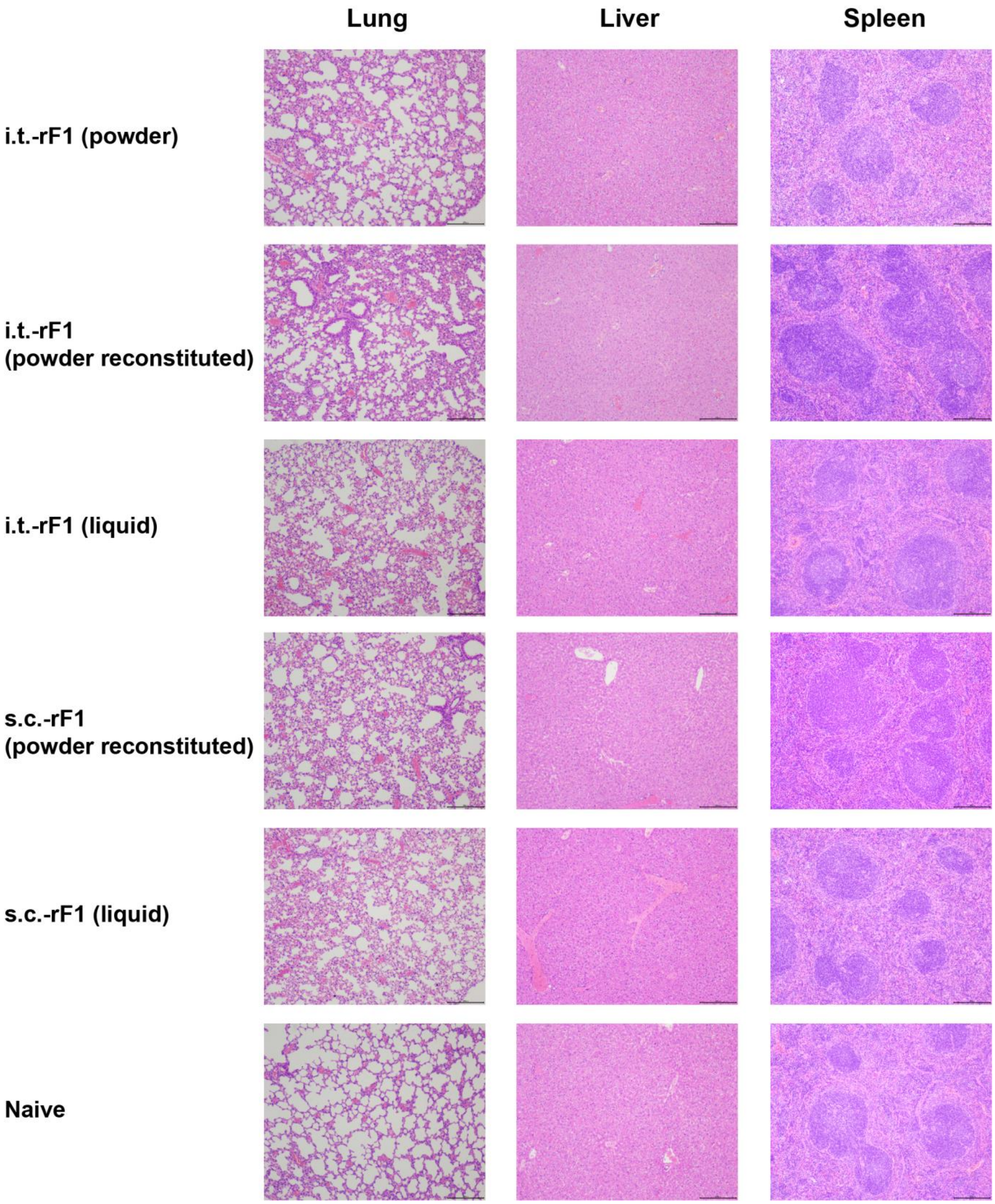
Lung
Liver
Spleen
i.t.-rF1 (powder)
i.t.-rF1
(powder reconstituted)
i.t.-rF1 (liquid)
s.c.-rF1
(powder reconstituted)
s.c.-rF1 (liquid)
Naive

B

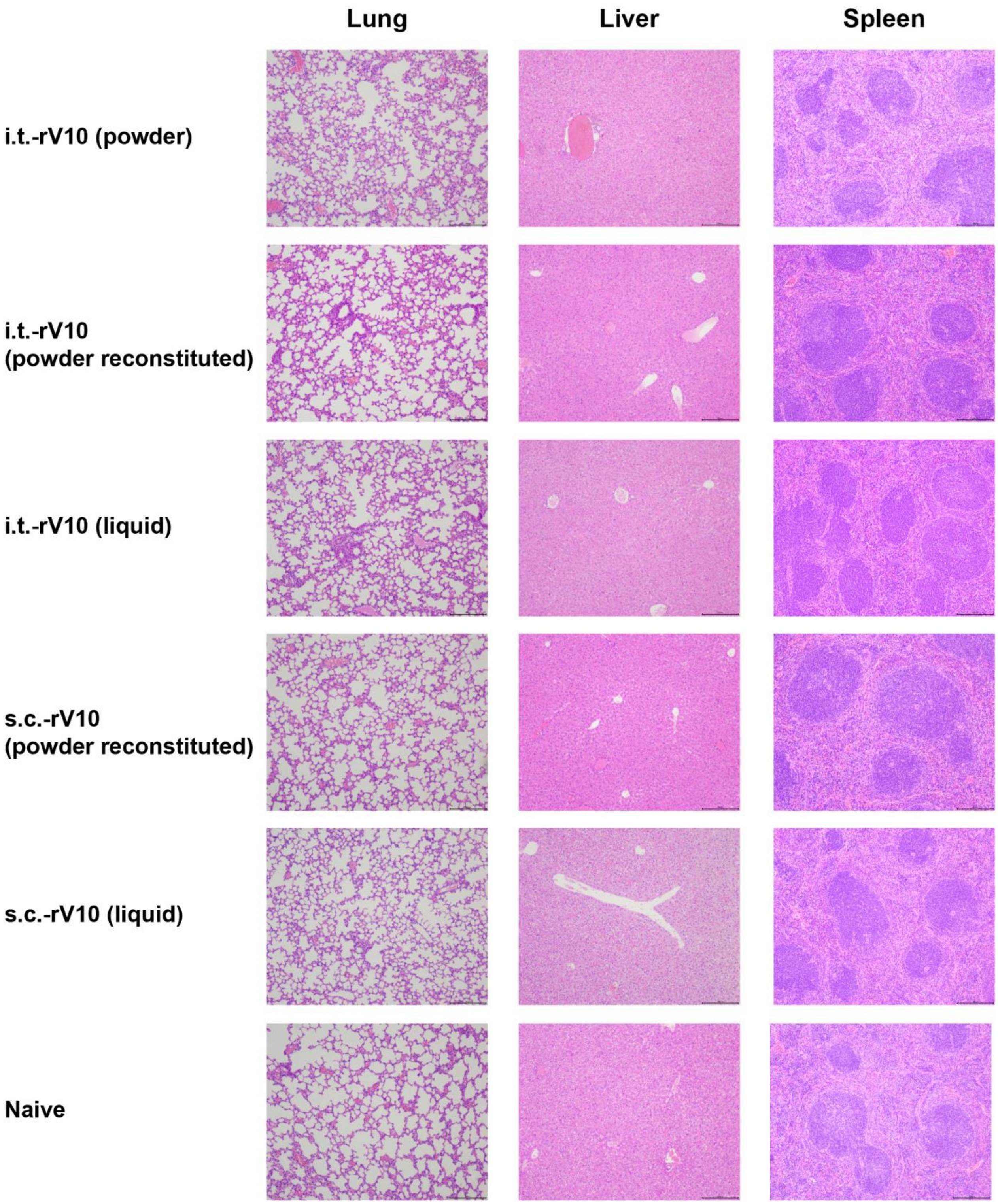
Lung
Liver
Spleen
i.t.-rV10 (powder)
i.t.-rV10
(powder reconstituted)
i.t.-rV10 (liquid)
s.c.-rV10
(powder reconstituted)
s.c.-rV10 (liquid)
Naive

C

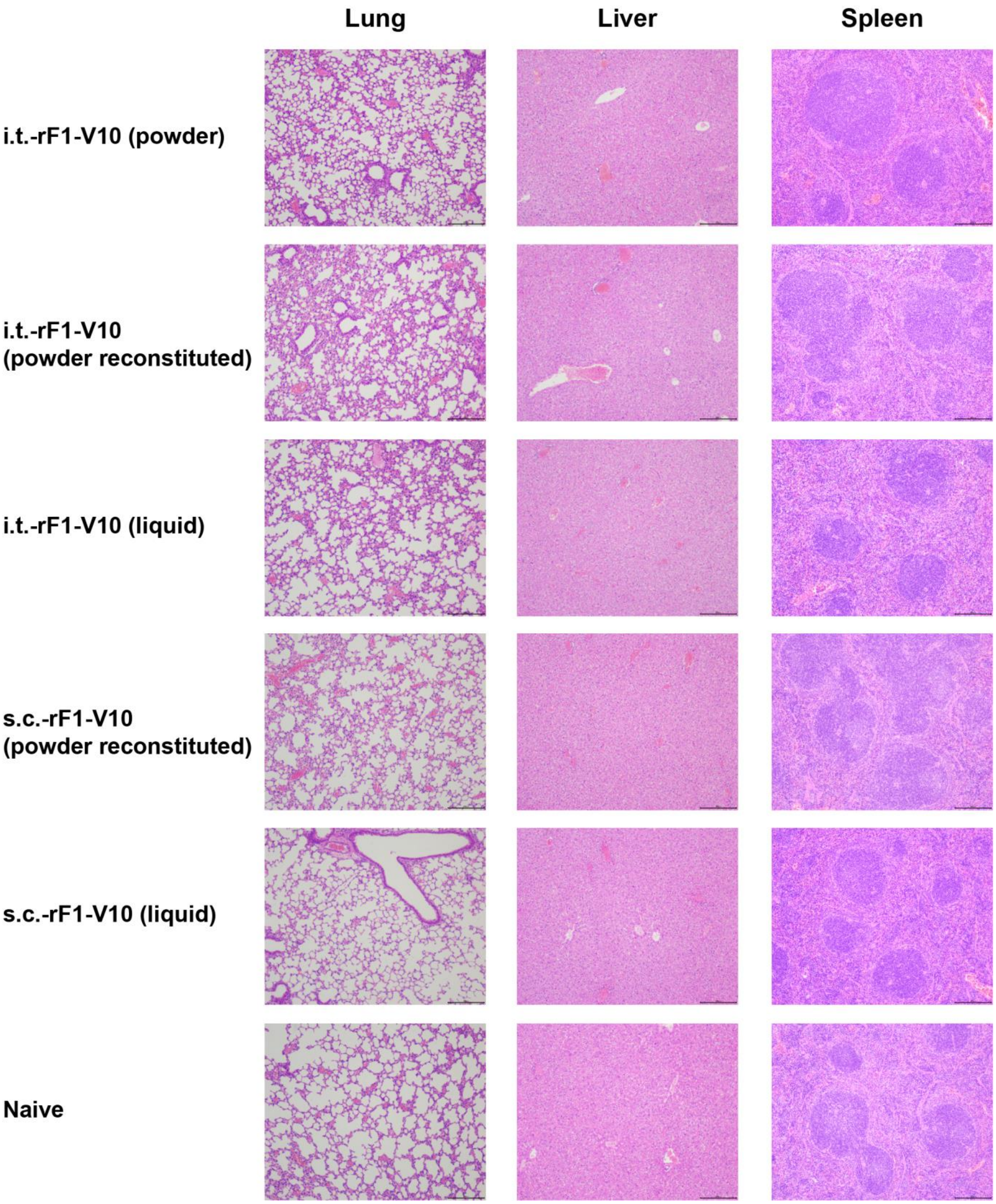
Lung
Liver
Spleen
i.t.-rF1-V10 (powder)
i.t.-rF1-V10
(powder reconstituted)
i.t.-rF1-V10 (liquid)
s.c.-rF1-V10
(powder reconstituted)
s.c.-rF1-V10 (liquid)
Naive

D

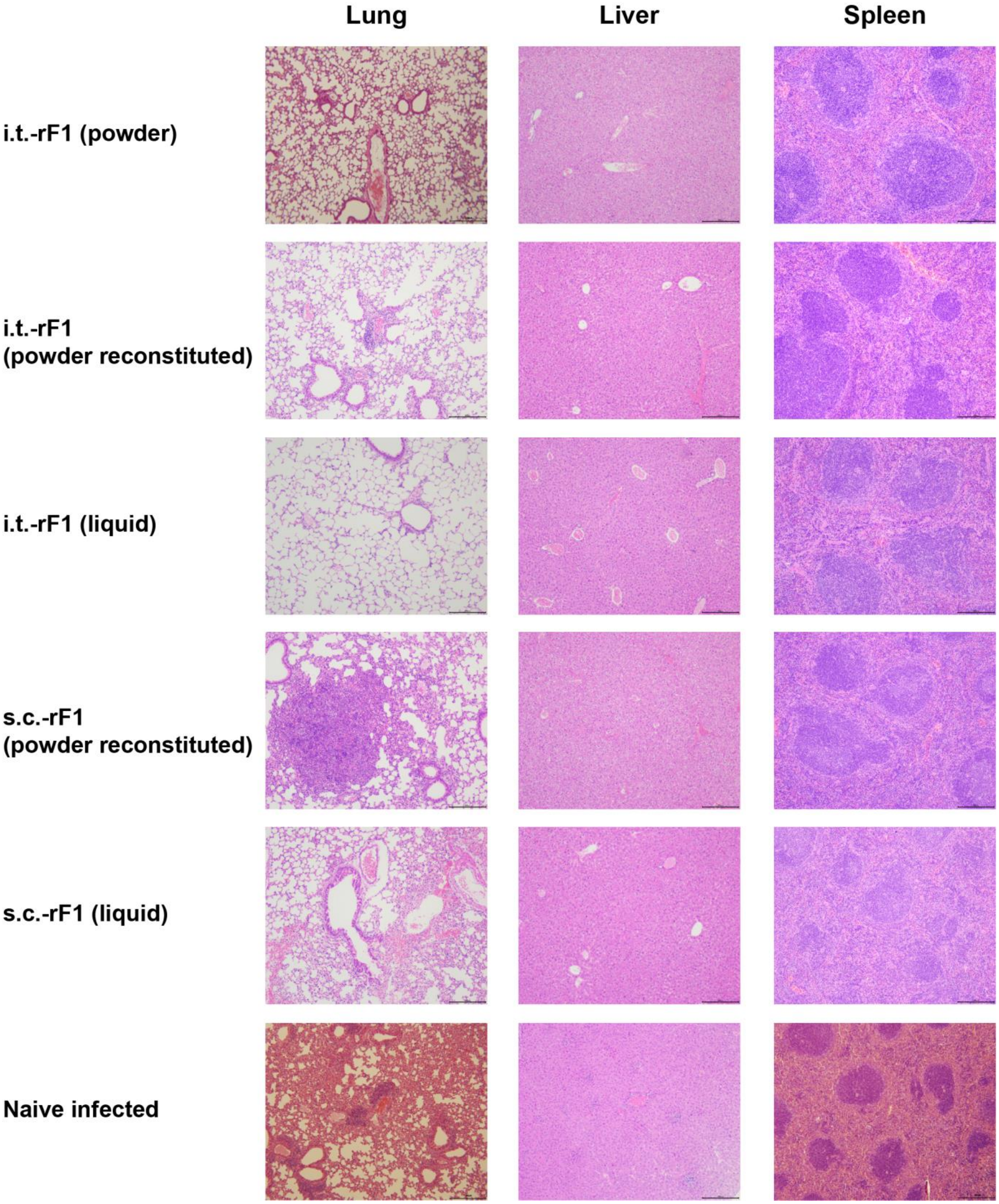
Lung
Liver
Spleen
i.t.-rF1 (powder)
i.t.-rF1
(powder reconstituted)
i.t.-rF1 (liquid)
s.c.-rF1
(powder reconstituted)
s.c.-rF1 (liquid)
Naive infected

E

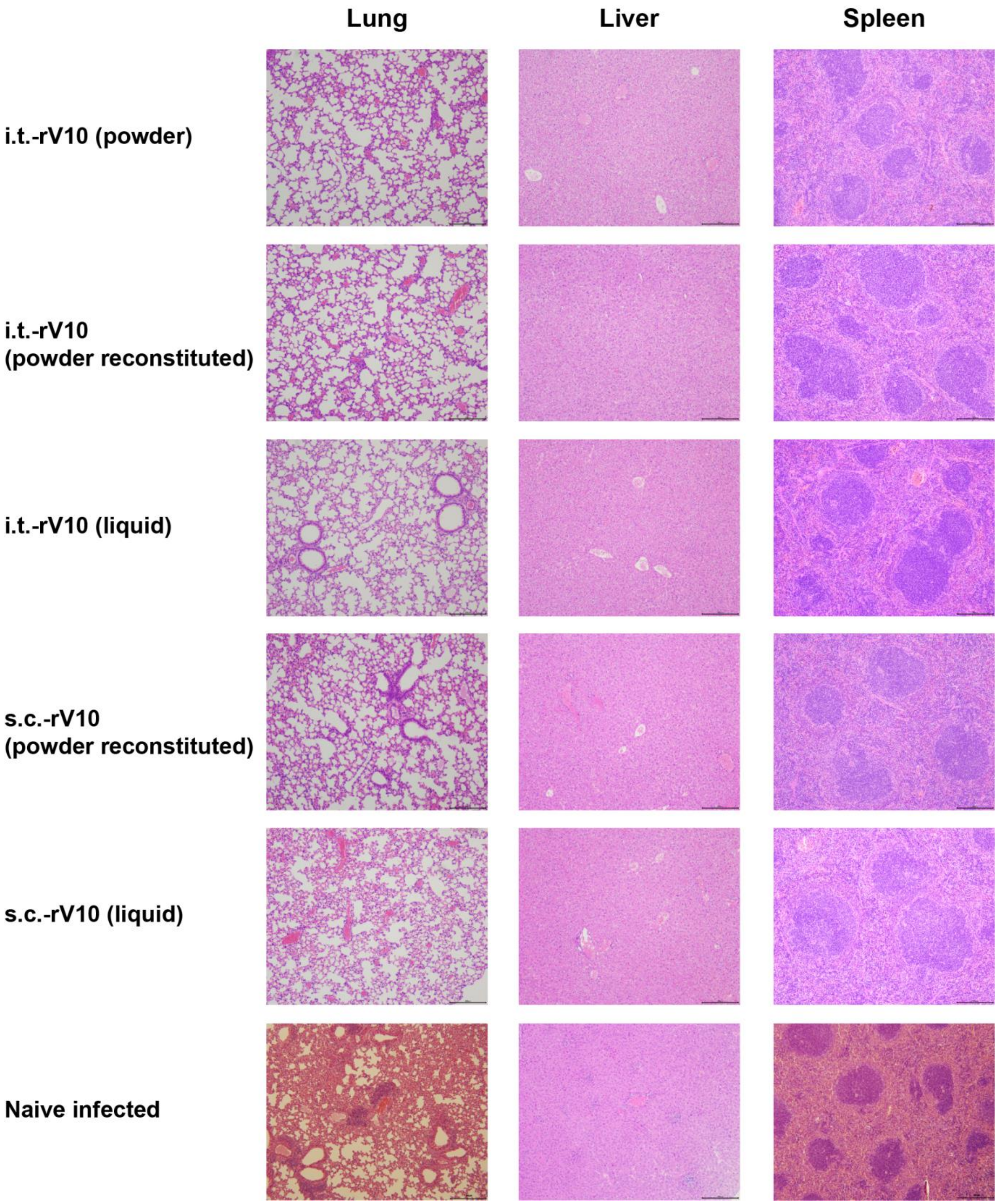

Lung
Liver
Spleen
i.t.-rV10 (powder)
i.t.-rV10 (powder reconstituted)
i.t.-rV10 (liquid)
s.c.-rV10 (powder reconstituted)
s.c.-rV10 (liquid)
Naive infected

F

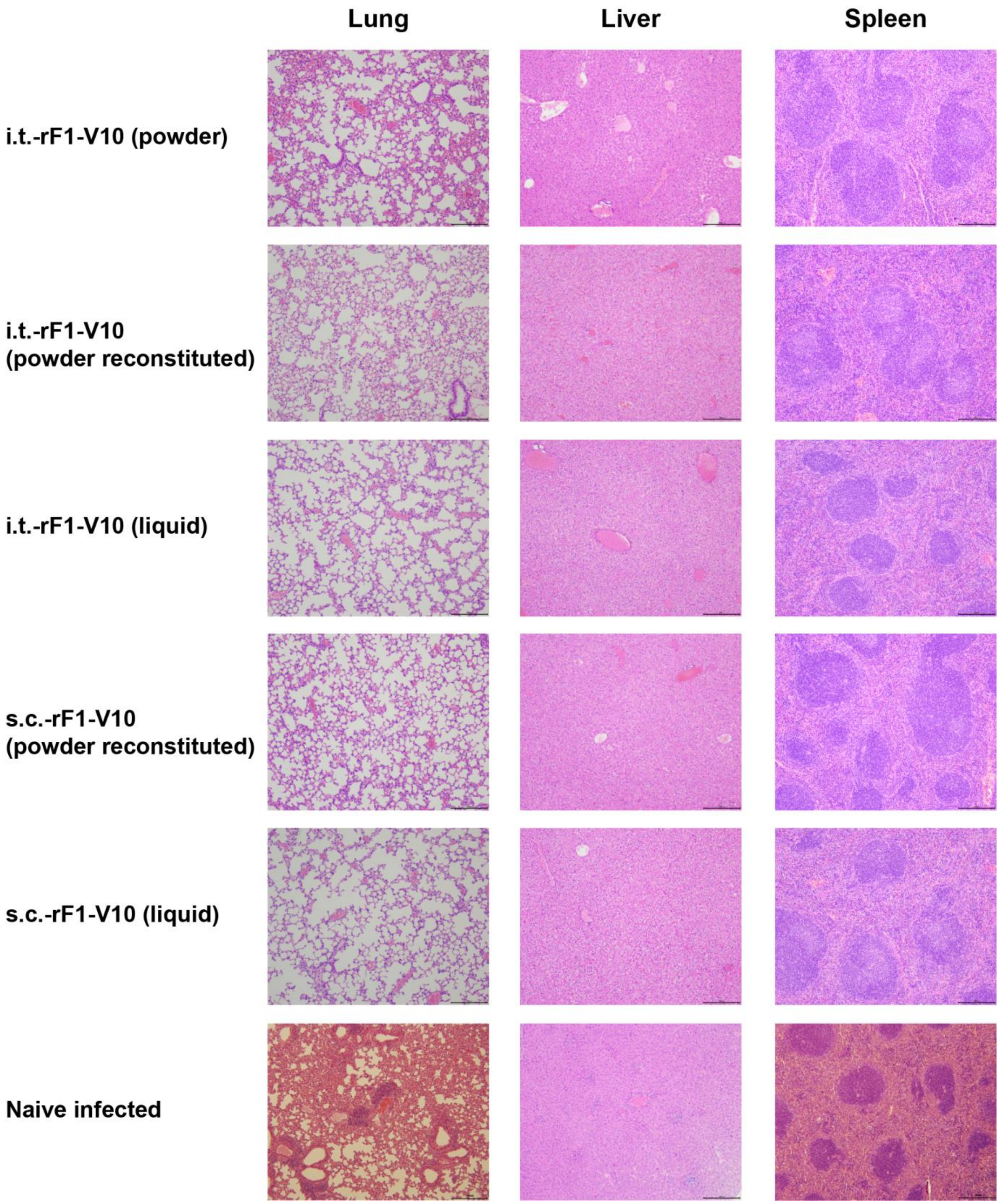

Lung
Liver
Spleen
i.t.-rF1-V10 (powder)
i.t.-rF1-V10
(powder reconstituted)
i.t.-rF1-V10 (liquid)
s.c.-rF1-V10
(powder reconstituted)
s.c.-rF1-V10 (liquid)
Naive infected

Supplementary Figure 2. Pathological changes in the tissues of mice after immunization and challenge. Three mice per group were euthanized after the third vaccination and 2 days post 50× LD_{50} i.t. challenge. Part of their lungs, livers and spleens were collected. **(A-C)** Tissues of mice after the third vaccination with **(A)** F1, **(B)** rV10, or **(C)** rF1-V10. Each vaccination type included five formulation x delivery method levels, plus an unvaccinated control. **(D-F)** Tissues from mice collected 2 days post-challenge and previously vaccinated with **(D)** rF1, **(E)** rV10, or **(F)** rF1-V10. Tissues were treated by conventional histopathological methods and HE stained sections examined by light microscopy. Tissue sections were evaluated by a trained pathologist according to the following scores: 0, no pathological lesions; 1, minimal; 2, mild; 3, moderate; 4, severe. The experiments were performed twice independently with similar results.
